# Organellar genomes of the four-toothed moss, *Tetraphis pellucida*

**DOI:** 10.1186/1471-2164-15-383

**Published:** 2014-05-19

**Authors:** Neil E Bell, Jeffrey L Boore, Brent D Mishler, Jaakko Hyvönen

**Affiliations:** Botanical Museum, Finnish Museum of Natural History, University of Helsinki, PO Box 7, FI-00014 Helsinki, Finland; Plant Biology, Department of Biosciences, University of Helsinki, PO Box 65, FI-00014 Helsinki, Finland; Department of Integrative Biology, University of California Berkeley, 1005 Valley Life Sciences Building, Berkeley, CA 94720-3140 USA; Department of Integrative Biology and University and Jepson Herbaria, University of California, 1001 Valley Life Sciences Bldg, Berkeley, CA 94720-2465 USA

**Keywords:** Moss, *Tetraphis pellucida*, Peristome, *nad7*, *petN*, Phylogeny, Organellar genomes, Sequencing

## Abstract

**Background:**

Mosses are the largest of the three extant clades of gametophyte-dominant land plants and remain poorly studied using comparative genomic methods. Major monophyletic moss lineages are characterised by different types of a spore dehiscence apparatus called the peristome, and the most important unsolved problem in higher-level moss systematics is the branching order of these peristomate clades. Organellar genome sequencing offers the potential to resolve this issue through the provision of both genomic structural characters and a greatly increased quantity of nucleotide substitution characters, as well as to elucidate organellar evolution in mosses. We publish and describe the chloroplast and mitochondrial genomes of *Tetraphis pellucida*, representative of the most phylogenetically intractable and morphologically isolated peristomate lineage.

**Results:**

Assembly of reads from Illumina SBS and Pacific Biosciences RS sequencing reveals that the *Tetraphis* chloroplast genome comprises 127,489 bp and the mitochondrial genome 107,730 bp. Although genomic structures are similar to those of the small number of other known moss organellar genomes, the chloroplast lacks the *petN* gene (in common with *Tortula ruralis*) and the mitochondrion has only a non-functional pseudogenised remnant of *nad7* (uniquely amongst known moss chondromes).

**Conclusions:**

Structural genomic features exist with the potential to be informative for phylogenetic relationships amongst the peristomate moss lineages, and thus organellar genome sequences are urgently required for exemplars from other clades. The unique genomic and morphological features of *Tetraphis* confirm its importance for resolving one of the major questions in land plant phylogeny and for understanding the evolution of the peristome, a likely key innovation underlying the diversity of mosses. The functional loss of *nad7* from the chondrome is now shown to have occurred independently in all three bryophyte clades as well as in the early-diverging tracheophyte *Huperzia squarrosa*.

## Background

Land plants (embryophytes) comprise three extant gametophyte-dominant clades (mosses, liverworts and hornworts) and one extant sporophyte-dominant clade (tracheophytes). The former comprise a paraphyletic grade known as the “bryophytes”, a morphological grouping of convenience that includes all lineages in which the diploid generation (the sporophyte) is unbranched, has only a single sporangium, and remains attached to a generally more complex, well-developed, and persistent haploid generation (the gametophyte).

Compared to the tracheophytes, there has been very little study of the organellar genomes of bryophytes. More genomic data are needed from representatives of each major clade, to solidify phylogenetic understanding as well as to investigate organellar evolution within the group. A key phylogenetic question that genomic data may be able to contribute towards solving is the branching order of the major peristomate clades (the *peristome* is the collective term for the elaborate teeth around the mouth of the capsule that provide a mechanism to control spore release). Sampling from the more isolated and morphologically distinct lineages surviving from this relatively early diversification event may also be an efficient strategy for uncovering genomic diversity of relevance to organellar evolution in land plants.

In contrast to liverworts, moss sporophytes exhibit considerable structural diversity, particularly in the morphology and function of the peristome. Although peristomes are lacking in the earliest-diverging moss lineages (Takakiopsida, Sphagnopsida, Andreaeopsida, and Andreaeobryopsida), they characterise all other major groups of mosses, representing about 97% of species diversity [1], except for the phylogenetically enigmatic Oedipodiopsida. Results from molecular systematic studies have corroborated the conclusion of early investigations (e.g., [2, 3]) that distinct peristome types define the primary phylogenetic divisions among peristomate mosses (e.g.; [4–8]).

**Figure 1:**
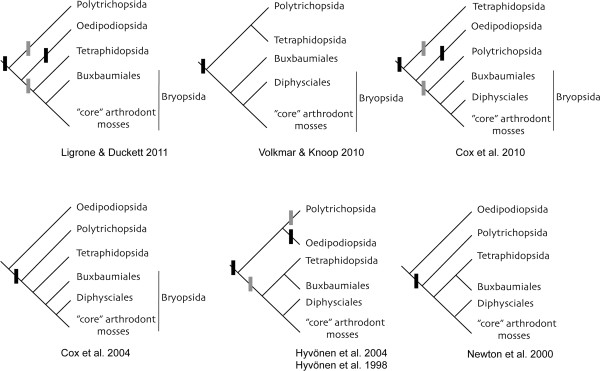
**Various topologies proposed for peristomate mosses in recent phylogenetic analyses.** Possible character state changes for gain and/or loss of peristomes with teeth are marked on trees with bars. In some cases alternative optimisations are indicated with lighter coloured bars.

Although each of these peristomate clades is clearly monophyletic (the Polytrichopsida, the Tetraphidopsida, the Buxbaumiales, and the *arthrodontous* clade, in which peristomes consist entirely of cell wall remnants), their relationships to each other and to the non-peristomate Oedipodiopsida is perhaps the most significant unsolved problem remaining in higher-level moss systematics (e.g., [4, 5, 9–13]). It has profound implications for hypotheses of homology among peristome types and whether peristomes have evolved more than once (Figure 1). Central to this problem is the position of the Tetraphidopsida, a small group of two genera and five species with a seemingly paradoxical combination of morphological and molecular traits.

Members of the Tetraphidopsida have peristomes unlike those in any other group of mosses, with the entire apical, peristome-forming portion of the sporophyte divided into four massive teeth. Based solely on the observation that these teeth are comprised of entire, elongated, thick-walled cells rather than cell wall remnants as in the arthrodontous mosses, the “nematodontous” Tetraphidopsida have traditionally been considered to be related to the Polytrichopsida, in which the very different peristomes also have these features. Developmentally, however, Shaw & Anderson [14] demonstrated that unlike in the Polytrichopsida, the peristome of *Tetraphis pellucida* is identical to that of arthrodontous mosses until a fairly late stage. Furthermore, the mature peristome in the Polytrichopsida is structurally very different from that of *Tetraphis*, and recent studies of relationships within the group strongly suggest that it is a derived feature, since the earliest diverging lineages lack peristome teeth altogether [13, 15]. All of these observations cast doubt on the idea that a generalized nematodontous peristome, homologous between Tetraphidopsida and Polytrichopsida, is plesiomorphic to the arthodontous peristome.

It seems plausible that peristomes (at least as structures composed of multiple linear processes remaining after dehiscence of the *operculum,* or capsule lid) might have arisen independently in the Polytrichopsida and in the common ancestor of the Tetraphidopsida and arthrodontous mosses (Figure 1), or even separately in all three lineages, perhaps from a primitive “pre-peristomate” structure. In this case it would be most parsimonious to interpret *Oedipodium* as also primitively non-peristomate, even if it is sister to the Tetraphidopsida, *Buxbaumia*, and the arthrodontous mosses [9] rather than to all of the peristomate mosses [10]. Clearly, however, the nearly identical and highly regular development of the peristome-forming cell layers in *Tetraphis*, *Buxbaumia* and the arthrodontous mosses, and to a lesser extent the Polytrichopsida [16, 17], is likely to be homologous, even if it did not originally gave rise to what we would now call a peristome.

In order to address these questions it is necessary to expand the quantity and quality of the phylogenetic characters available to inform relationships among the major peristomate groups, and in particular to clarify the position of the Tetraphidopsida. Sequencing of complete organellar (plastid and mitochondrial) genomes offers the potential to considerably increase the number of nucleotide characters and to provide new genomic-level characters, as well as to elucidate organellar evolution. Potential genomic characters include gene presence/absence, gene inversions, intron insertion and deletion, pseudogenisation processes, and changes in gene order, and can be viewed as morphological characters at the genetic level [18–22]. Currently there are still only two complete chloroplast genomes fully published for mosses, for *Physcomitrella patens*[23] and *Tortula ruralis*[24], although plastid genomes have been sequenced for a number of other species including *Takakia lepidozioides*, *Sphagnum palustre*, and *Andreaea nivalis*, with genomic level characters (mainly gene losses and pseudogenisation) being found that are congruent with currently accepted relationships [25]. Similarly, there are only two complete mitochondrial genomes published for mosses at time of writing, for *P. patens*[26] and *Anomodon rugelii*[27], although genomes from a range of mosses have been assembled, with the composition reported as highly conserved [28]. As a step towards providing genomic level data to address the question of relationships among the peristomate moss groups, we sequenced and fully assembled the chloroplast and mitochondrial genomes of *Tetraphis pellucida* using a mixture of Illumina SBS and Pacific Biosciences (PacBio) RS sequencing. The results provide preliminary insights into phylogeny and organellar evolution, which we anticipate will be developed further when similar data becomes available for other key lineages.

## Results

### Chloroplast genome

**Figure 2:**
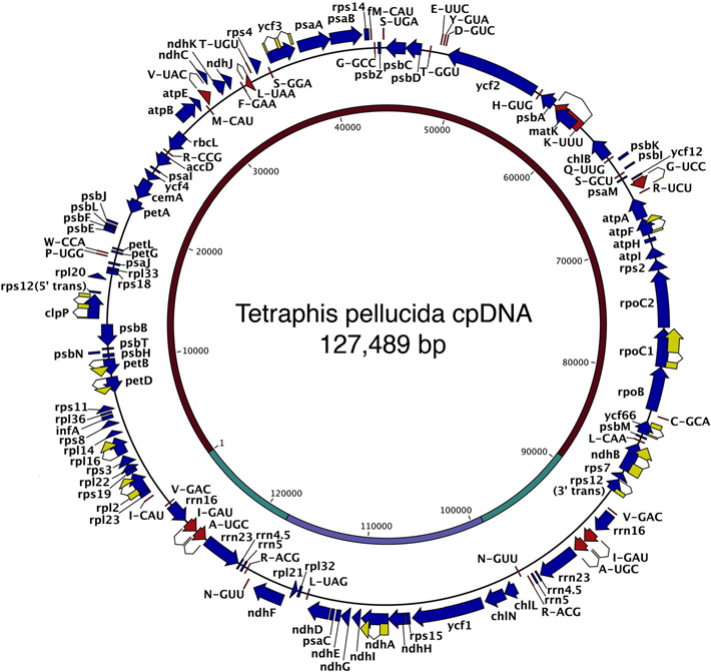
**Gene map of the** ***Tetraphis pellucida*** **chloroplast genome.** Protein-encoding and rRNA-encoding genes are in blue and tRNA-encoding genes are in red. All genes reading clockwise are shown, with their names, outside of the circle. All genes reading counter-clockwise are shown on the circle with their names inside. All genes with multiple exons have these shown in yellow flanking the gene names. Single letters designate tRNA genes according to the one-letter code for the corresponding amino acid with “fM” indicating the tRNA expected to be charged with formyl-methionine. tRNA-encoding genes are further differentiated with the codon expected to be recognized in cases where there are more than one tRNA for the same amino acid. As is common, *rps12* occurs in two non-contiguous regions that are spliced *in trans* to form a complete transcript, so these are shown with the words “5′ trans” and “3′ trans” to indicate this. The inner circle shows the large single-copy (LSC) region and small single-copy (SSC) region in brown and blue, respectively, and the inverted repeat regions in green, with nucleotides numbered starting at the beginning of the LSC.

The chloroplast genome of *Tetraphis pellucida* has a length of 127,489 bp and retains the general structure common to most land plants, with two inverted repeat (IR) regions of 9,564 bp separated by a small single copy region (SSC) of 18,927 bp and a large single copy region (LSC) of 89,434 bp. Overall GC content is 29.4%, similar to other known bryophyte chloroplast genomes (28-33% [23]) and significantly less than the 34-40% found in seed plants [29]. The IR gene content is identical to that of the mosses *Tortula ruralis* and *Physcomitrella patens*, with the *trnV*-GAC and *trnN*-GUU transfer RNA genes terminating the IRs. Figure 2 illustrates the structure of the genome and the relative positions of all genes.

In common with *Tortula ruralis*, *Tetraphis pellucida* lacks the sizeable inversion of around 71 kb in the LSC that characterizes *P. patens* and other Funariales [30]. It also shares with *Tortula ruralis* the absence of *petN*, these two species being the only land plants currently known to lack this gene in the chloroplast. Otherwise, functional gene content and order are identical in all three of these mosses. The assembly also corroborates the absence of the *rpoA* gene, previously reported to have been absent in *Tetraphis* as well as in all arthrodontous groups (including *Diphyscium*), although present in all other major moss lineages, including *Buxbaumia*[31].

In *Tortula ruralis* a pseudogenised copy of the *trnP*-GGG gene was reported, but a nucleotide BLAST (BLASTn) of the corresponding area in the *Tetraphis pellucida* assembly matched only a small part of the spacer region adjacent to this pseudogene in *Tortula ruralis*. Similarly, while a *tufA* pseudogene is present in the *Takakia* chloroplast genome (accession number AB367138, incomplete assembly), a BLASTn of the corresponding *Tetraphis pellucida* DNA did not match this.

Despite identical or near identical gene content, the *Tetraphis pellucida* chloroplast genome is approximately 5 kb longer than those of *Tortula ruralis* (122,530) and *P. patens* (122,890). This difference is nearly entirely accounted for by an increased total length of intragenic spacer regions in the LSC (see Table 1 for basic compositional statistics).

A scan for candidate RNA editing sites (C => U) using the protein sequence BLAST prediction method (BLASTx) implemented in PREPACT 2.0 [32] found 15 potential sites predicted by 100% of references and 25 potential sites predicted by 75% of references. Only one of the sites predicted by 75% or 100% of references corresponded to a previously identified RNA editing event in a reference (in *psbB* in *Anthoceros formosae*, label psbBeU38PL). A scan of the published *Tortula ruralis* chloroplast genome for comparative purposes yielded only three potential sites predicted by 100% of references and 10 predicted by 75% of references, with none of these corresponding to previously identified editing events.

### Mitochondrial genome

**Figure 3:**
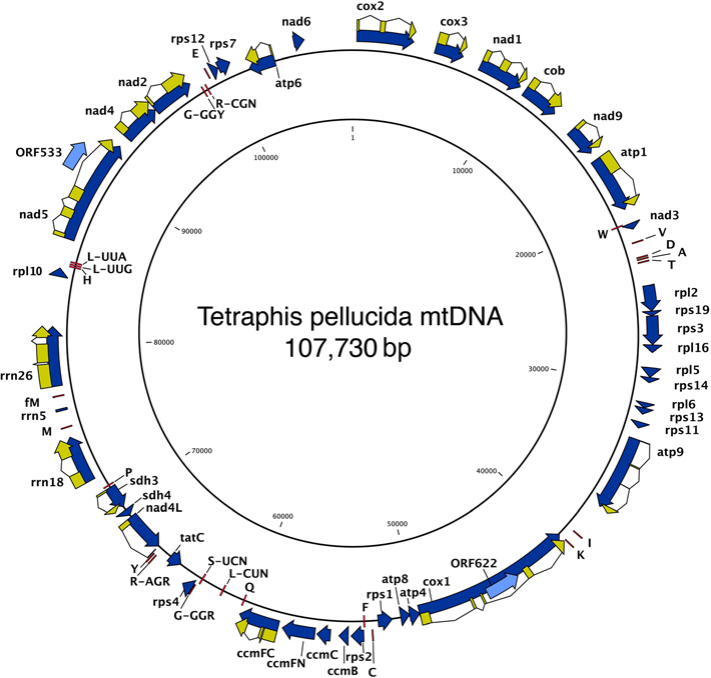
**Gene map of the** ***Tetraphis pellucida*** **mitochondrial genome.** Genes are shown as explained for Figure 2.

The *Tetraphis pellucida* mitochondrial genome (structure illustrated in Figure 3) has a total length of 107,730 bp and an overall GC content of 42.5% (see Table 1 for statistics). Both of these figures are similar to, if slightly higher than, those for *P. patens* (105,340 bp, 40.6% GC; [26]) and *Anomodon rugelii* (104,239 bp, 41.2% GC; [27]). Gene order is identical to that of the other two known moss chondromes, but the *nad7* gene, which is present as a functional gene in the published gene maps and GenBank annotations for *Physcomitrella patens* and *A. rugelii*, is present only as a partial pseudogene in *T. pellucida*. The region between the *trnT* and *rpl2* genes in *T. pellucida* consists of only 1,329 bp, with BLASTn results for this area indicating 81% and 86% alignment to sections of the *nad7* genes of *A. rugelii* and *P. patens* respectively and 90% sequence identity in both cases. However, in both *A. rugelii* and *P. patens* the *nad7* gene occurs in three exons of 140 bp, 69 bp and 973 bp separated by two large introns, while in *T. pellucida* the largest downstream exon and most of the preceding intron are entirely absent. Also absent in *T. pellucida* is the pseudogenised *rps10* gene, present in *A. rugelii* and *P. patens* downstream of *nad7* and preceding the start of the *rpl2* gene*.* Nonetheless the two shorter *nad7* exons and intervening intron are present and alignable with those of *A. rugelii* and *P. patens*, the exons apparently with uninterrupted reading frames.

In *T. pellucida* there is only a short region of 283 bp between the second 69 bp exon of the pseudogenised *nad7* and the *rpl2* gene. Most of the first 161 bp is alignable with the start of the downstream intron in the *nad7* genes of *A. rugelii* and *P. patens*, but the final 122 bp return only a single match in a BLASTn search (87% identity), to the same region immediately upstream of the *rpl2* gene in the lycopod *Huperzia squarrosa*, a species in which the *nad7* gene is also functionally absent [33].

The pseudogenised copy of *rps8* occurring next to the *rpl6* gene in *A. rugelii* and *P. patens* is identifiable also in *T. pellucida*.

Scanning for RNA editing sites (C => U) using the BLASTx prediction method in PREPACT 2.0 found 62 potential sites predicted by 100% of references and 89 potential sites predicted by 75% of references. Of these, 45 and 54 respectively corresponded to previously identified RNA editing events in at least one reference.

**Table 1:** **Selected statistics for chloroplast and mitochondrial genomes of** ***Tetraphis pellucida***

	Chloroplast	Mitochondrion
Total length (bp)	127,489	107,730
Protein coding (genes/ORFs [exons bp total])	82/83 [69,897]	41/41 [36,285]*
Ribosomal (genes [exons bp total])	8 [4,532]	3 [4,822]
tRNA (genes [exons bp total])	36 [2,705]	24 [1,795]
Other (introns, spacers, pseudogenes etc., bp total)	50,355	64,828
Adenine, A (bp [%])	45,073 [35.4%]	31,162 [28.9%]
Cytosine, C (bp [%])	18,834 [14.8%]	22,098 [20.5%]
Guanine, G (bp [%])	18,635 [14.6%]	23,720 [22%]
Thymine, T (bp [%])	44,941 [35.3%]	30,750 [28.5%]
Weight (single-stranded)	39.371 MDa	33.313 MDa
Weight (double-stranded)	78.749 MDa	66.558 MDa

*All counts include the two unidentified ORFs (see Figure 3).

## Discussion

The chloroplast genome of *Tetraphis pellucida* is identical in overall structure, gene content, and gene order to that of *Tortula ruralis* and highly similar to that of *Physcomitrella patens*, while the mitochondrial genome is identical in gene order to those of *Anomodon rugelii* and *P. patens*, except differing from both in the absence of a functional *nad7* gene. The latter is due to the absence of a region that includes the largest exon of *nad7* as well as the pseudogenised *rps10* gene. Although the full value of the data for phylogenetic reconstruction and the study of organellar evolution within the mosses will not become apparent until genomes from other major moss lineages are fully assembled and published, a number of observations can be made at this stage based on comparison with data that are already available.

Predicted levels of organellar RNA editing in *Tetraphis pellucida* are consistent with a hypothesis of a general trend towards reduction in numbers of edited sites in more derived (or at least later diverging) clades within the mosses, with numbers of both chloroplast and mitochondrial sites being somewhat higher than those found in arthrodontous mosses and numbers of chloroplast sites very much lower than those found in the early-diverging moss lineage *Takakia* as well as in hornworts [34–37]. Yura et al. [34] found 302 putative RNA editing sites in the chloroplast genome of *Takakia lepidozioides*, while Miyata et al. [36] found only two in *P. patens* and our own results predict three in *Tortula ruralis* (an arthrodont, like *P. patens*). This compares with our predicted figure of 15 sites for *Tetraphis pellucida*. Similarly, our predicted figure of 62 mitochondrial RNA editing sites for *Tetraphis pellucida* is considerably higher than the 11 sites present in *P. patens*[37] and the 39 predicted for *Anomodon rugelii* by Lenz & Knoop [32].

The pseudogenisation of *nad7* in the *Tetraphis pellucida* chondrome is significant, as while it is also absent or existing only as a non-functional pseudogene in hornworts and in most liverworts [38–40] as well as in the lycopod *Huperzia squarrosa*[33], it appears to be functionally present in all other known tracheophyte and moss chondromes investigated, including representatives of *Takakia*, *Ulota*, *Leucobryum* and *Dichodontium*[41] as well as *P. patens* and *A. rugelii*. In liverworts, both *Haplomitrium*[38] and *Treubia*[27] retain a functional mitochondrial *nad7* gene, while all other species investigated have pseudogenised copies, varying greatly in their degrees of degeneration but remarkable for having persisted throughout the long evolutionary history of the group [38]. This is consistent with current hypotheses of *Haplomitrium* and *Treubia* forming the sister clade (Haplomitriopsida) to the rest of the liverworts (Marchantiopsida and Jungermanniopsida, e.g. [42, 43]) and the psuedogenisation of *nad7* having occurred in a common ancestor of the extant Marchantiopsida and Jungermanniopsida. In *Marchantia polymorpha* at least, there is clear evidence for endosymbiotic gene transfer to the nucleus and a functional copy of *nad7* in the nuclear genome [44], and it can reasonably be assumed that this is the case for all other non-Haplomitriopsid liverworts. Interestingly, the functional *nad7* gene found in other mosses shares its two introns with the version found in vascular plants [41], while in the liverwort *nad7* gene these are lacking, and two other non-homologous introns occur instead [41, 38]. The current finding that *nad7* is present only as a pseudogenised remnant in the *T. pellucida* chondrome demonstrates that loss of a functional mitochondrial copy of this gene has occurred independently in all three bryophyte clades as well as in at least one early-diverging tracheophyte (*Huperzia*). However, while this appears to have been a very early and defining event in the evolution of both hornworts and liverworts, it is a derived condition in mosses and conceivably even unique to *Tetraphis* (as it may be unique to *Huperzia* within the tracheophytes). Wahrmund et al. [45] showed that the entire *trnA-trnT-nad7* gene cluster has been subject to extensive recombination during the evolutionary history of liverworts. However, it is striking that a BLASTn search for the region of 122 bp adjacent to the *rpl2* gene in *T. pellucida* matches only the same region in *H. squarrosa*, suggesting the possibility of a shared mechanism for the independent loss of *nad7* in *Tetraphis* and *Huperzia*. Unlike *T. pellucida* however, *Huperzia* appears to lack any traces of the two smaller *nad7* exons (which are present with uninterrupted reading frames in *Tetraphis*), suggesting that pseudogenisation following the loss of the large exon is considerably more advanced in *Huperzia*.

Although the role of *nad7* in encoding a subunit of respiratory complex I (RC 1, NADH-ubiquinone oxidoreductase) might imply that it is indispensable, in plants the existence of alternative NAD(P)H-ubiquinone oxidoreductases means that mutants with a deficient RC I are potentially viable [46]. In the tobacco mutant CMSII, which lacks RC I activity, this has been shown to be due to deletion of the *nad7* gene from the mitochondrion [46]. Although these plants exhibit slow development and reduced vegetative and floral organs they are apparently viable in cultivation. Although it is much more probable that in *T. pellucida* the *nad7* gene has been transferred to the nuclear genome (as in *Marchantia*) than that *nad7* and/or RC I activity is lacking, perhaps the relative simplicity and slow growth rates of bryophytes make endosymbiotic transfer of this gene more likely to occur, because temporary loss of RC I functionality might have less chance of being immediately fatal.

Of further note is the absence of *petN* in the plastome of *Tetraphis pellucida*, while this gene is present in *Physcomitrella patens*[23], as well as in all other known embryophytes except *Tortula ruralis*. Unpublished data additionally suggest that *petN* is present in at least *Sphagnum palustre*[25]. On noting the absence of *petN* in *Tortula ruralis*, Oliver et al. [24] considered it most probable that it has been transferred to the nuclear genome, or else that another nuclear-encoded gene product performs the same function as a subunit of the photosynthetic cytochrome b6f complex. The shared absence of *petN* in *Tortula* and *Tetraphis* and its presence in *Physcomitrella* and other Funariales [47] requires either that a loss has occurred more than once, that *petN* has been regained in the Funariales (which seems improbable), or that *Tetraphis* (or the *Tetraphis* chloroplast) is more closely related to *Tortula* than *Tortula* is to *Physcomitrella*. As both *Physcomitrella* and *Tortula* are unambiguously arthrodontous, highly supported by phylogenetic analyses of nucleotide data, the latter also seems unlikely and is at odds with all recent phylogenetic analyses (Figure 1). Both the unique structure of the tetraphidopsidan peristome (see Background) and existing molecular phylogenetic studies strongly suggest that the Tetraphidopsida are outside of the major clade of arthrodontous mosses, while generally also implying that *Buxbaumia* is more closely related to the arthrodonts than *Tetraphis* is. Further studies of presence/absence of *petN* in moss plastomes are needed.

The *rps16* gene appears to be plesiomorphically present in the plastomes of exemplars of non-peristomate moss lineages [25], including *Andreaea nivalis,* representative of the Andreaeopsida (the sister lineage to the peristomate mosses + *Oedipodium*), although it is absent in *Physcomitrella* and *Tortula*. The current results show that it is also absent in *Tetraphis pellucida*, which is consistent with a loss in a common ancestor of the Tetraphidopsida and the arthrodontous mosses, originating after divergence with the Andreaeopsida. Thus the presence of this gene in the plastome has the potential to be phylogenetically informative for relationships amongst the major peristomate lineages, depending on which other groups (Oedipodopsida, Polytrichopsida, and Buxbaumiales) it occurs in. Further adding to the pattern of gene presence/absence, it is known that the *rpoA* gene is present in all earlier diverging moss lineages (including *Buxbaumia* and the Polytrichopsida) but absent in all lineages of arthrodontous mosses as well as in *Tetraphis*[31].

Despite consensus on the monophyly of the arthrodontous mosses, molecular sequence comparisons have failed to consistently support any hypothesis of a relationship between the Tetraphidopsida and any other major group e.g. [4, 5, 10], (Figure 1). Thus after failing to corroborate the results of Cox et. al [10], in which *Tetraphis pellucida* was placed as sister to *Buxbaumia* plus the arthrodontous mosses, Cox et al. [4] concluded that (in relation to nematodontous peristomes) “the origin of the arthrodontous peristome”… “remains obscure”. Ligrone & Duckett [9] instead stressed putatively conserved morphological and molecular characters to support a phylogenetic hypothesis in which the Tetraphidopsida is sister to a clade comprising *Buxbaumia* and the arthrodontous mosses including *Diphyscium* (as in [10]), but with *Oedipodium* sister to this group rather than to a larger peristomate clade including the Polytrichopsida. The Polytrichopsida lack a distinctive placental morphology in the gametophyte/sporophyte junction that is found in all other peristomate mosses as well as in *Oedipodium*. The authors suggested that a nematodontous peristome arose once in the ancestor of all peristomate mosses and was lost in *Oedipodium*, while being retained in the Tetraphidopsida and also giving rise to the arthrodontous peristome. As discussed above, however, all hypotheses implying that the tetraphidopsidan peristome inherits features from a common ancestor shared with the Polytrichopsida are questionable, given that the Polytrichopsida appear to be primitively non-peristomate [13, 15].

It is likely that the evolutionary innovation represented by the development of peristomes in mosses was associated with a period of rapid lineage diversification, and that the time period separating the origin of the most recent common ancestor of all extant peristomate lineages from that of each individual lineage was short relative to the age of these events. Based on a phylogenetic reconstruction in which *Oedipodium* was resolved as sister to the peristomate mosses, Newton et al. [1] estimated the age of the node representing this initial split at 291 MYA (late Carboniferous), with a final split between *Buxbaumia* and the arthrodontous mosses estimated to have occurred in the mid Permian (approximately 275 MYA). The inability of phylogenetic analyses using nucleotide substitution data to satisfactorily resolve the sequence of branching events among the peristomate lineages may be due to multiple changes in most nucleotide characters evolving rapidly enough to potentially have been informative within this narrow hypothesised 15 MY window. In such cases, morphological characters representing significant structural innovations may be particularly useful because their rate of evolution may increase during periods of rapid diversification and decrease during periods of evolutionary stasis [48]. Similarly, it is possible that genomic rearrangements may be more frequent during periods of increased speciation, as suggested by accelerated rates of chondrome evolution in parasitic arthropods (e.g., [49–51]). Certainly, as relatively rare events they should be less subject to homoplasy than point mutations of nucleotides and thus particularly useful for phylogeny reconstruction [19, 20].

Unfortunately, organellar gene losses (if assumed to be associated with transference of function to the nucleus) may be the least reliable of such characters due to a relatively high potential for convergent evolution. We would expect transfer of an indispensable gene to the nucleus to occur prior to loss from the organellar genome, followed by the acquisition of regulatory signals for the gene and plastid import signals for the protein. If the initial transfer occurred in the common ancestor of a large clade, subsequent loss from the plastid would be significantly favoured in descendent lineages, but with a large element of chance governing when and in which lineages it occurred. Although such considerations caution against placing undue emphasis on gene loss events in isolation, such data nonetheless provide valuable characters that must be interpreted in the context of any given phylogenetic hypothesis as well as all other characters.

The functional absence of *nad7* from the chondrome of *Tetraphis pellucida* is currently uninformative phylogenetically, as no other mosses are known to share this feature. However, if a similar loss was found in representatives of one or more of the other lineages that have not yet been investigated for this gene, such as *Buxbaumia* or *Oedipodium*, the character could be highly informative for branching order of the major peristomate groups. Otherwise, the structure of the mitochondrial genome appears to be highly conserved amongst peristomate mosses, based on the three (relatively phylogenetically distant) exemplars for which fully assembled chondromes are available. It is possible that the phylogenetic utility of mitochondrial genome sequences in mosses may lie principally in nucleotide-level data. Conserved protein coding genes from the mitochondrion, such as *nad5*, have proven to be highly useful for phylogeny reconstruction at relatively deep nodes in mosses, such as at the ordinal level within subclass Bryidae [52–54].

The distribution of the chloroplast *rpoA* gene [31] suggests an unconventional phylogeny if a single state change is assumed, although not an entirely incredible one. Although Goffinet et al. [31] assumed a topology in which *Buxbaumia* is sister to *Diphyscium* and the other arthrodontous mosses and then reconstructed an independent loss of *rpoA* in *Tetraphis* and in the arthrodonts using likelihood (consistent with DELTRAN optimisation under parsimony), if *Tetraphis* is sister to the arthrodonts and *Buxbaumia* sister to that clade in turn, it would be necessary to assume only a single loss. Although the peristomes of *Buxbaumia* and *Diphyscium* are superficially highly similar and Shaw et al. [17] demonstrated that *Diphyscium* is effectively entirely arthrodontous in its peristomial structure, no such detailed developmental study exists for *Buxbaumia*, which has a considerably more developed “parastome”, apparently with some teeth comprised of entire cells (nematodontous) and involving exothecial layers outside of the OPL [55]. *Buxbaumia aphylla* also has more columns of cells in its peristomal layers (*Diphyscium* has 16 PPL cells and 32 OPL cells at maturity, as in most arthrodonts [17]) and a more irregular peristomal structure, further distancing it from the arthrodontous mosses. Assuming that *Tetraphis* is highly autapomorphic it is conceivable that it could be derived from an ancestor having the shared peristomal features of *Buxbaumia* and *Diphyscium*. Nonetheless, a significant number of characters link *Buxbaumia* to *Diphyscium* and the arthrodonts (including a characteristic deletion in the *rps4-trnA* intergenic spacer [10, 56]), and Goffinet at al.’s [31] proposed scenario currently seems most credible.

## Conclusions

Both the chloroplast and mitochondrial genomes of *Tetraphis pellucida* exhibit structural features that have the potential to be informative for reconstructing the branching order of the peristomate moss lineages. In order to exploit these data, fully assembled organellar genomes are urgently required from representatives of the other major groups (*Buxbaumia*, *Oedipodium*, and the Polytrichopsida). In particular, presence/absence of the mitochondrial *nad7* gene and the chloroplast *petN* gene (and potentially also the chloroplast *rps16*) will be of paramount importance when considered together with that of the *rpoA* chloroplast gene, which is better known at present. Although homoplasy (probably in the form of multiple losses of individual genes rather than reversals) is quite possible, when all of these characters are available for all lineages and can be analysed together, a single scenario, or a much smaller set of scenarios, may emerge as uniquely credibly supported. Furthermore, simultaneous phylogenetic analysis of the full complement of alignable nucleotide data from both organellar genomes may retrieve sufficient signal to resolve the ambiguities in the relatively small volume of such data that currently exists. Whether *Tetraphis* eventually is revealed to be a unique experiment in moss sporophyte dehiscence or as providing clues to stages in the evolutionary development of the arthrodontous peristome, understanding the precise relationships of this enigmatic moss to other extant taxa will be a significant piece in the puzzle of land plant evolutionary history.

## Methods

### DNA isolation, sequencing, and assembly

Material of *Tetraphis pellucida* was collected from two wild populations situated within 15 m of each other, both on rotten wood in dense, mixed coniferous-deciduous forest in Espoo, Finland (approximately 60°19′10″N, 24°30′00″E). Voucher specimens (Bell 03.03.10.001, Bell 03.03.10.002) are held in the herbarium of the Botanical Museum, Finnish Museum of Natural History (H). Two DNA extractions were made using different methods, both including material from both collections due to the large volume of material required (relative to the size of the plants) by one of the extraction methods. Plants of *T. pellucida* are small and sometimes grow mixed with other organisms, thus single shoots were separated under the dissecting microscope over several days with each shoot checked individually for identity and for contaminants visible at 40× magnification.

For Illumina SBS sequencing, a modified CTAB extraction protocol was used [57], incorporating an initial differential centrifugation step in an attempt to concentrate chloroplast DNA in the final preparation. A volume of shoots weighing 0.4 g was ground in liquid nitrogen and mixed by inversion in 20 ml of a buffer containing 50 mM Tris–HCl pH 8.0, 10 mM EDTA, 20% sucrose, 5 mM 2-mercaptoethanol and 0.1% BSA (from [58]). This suspension was passed through five layers of fine mesh cloth and then centrifuged for 30 minutes in a 50 ml centrifuge tube at 1000 rpm to remove unbroken cells and any remaining leaf and shoot fragments. The supernatant was centrifuged at 5000 rpm for 30 minutes to produce a (theoretically) chloroplast-rich pellet, this subsequently being used for CTAB extraction. However, later analysis of sequencing reads suggested that chloroplast DNA was not significantly increased in the final extraction by this method relative to DNA from other genomic compartments. For PacBio RS sequencing, a standard genomic extraction was prepared with a much smaller starting volume (approximately 10 mg) of material from both collections using the Invisorb spin plant mini kit (Invitek, Berlin, Germany).

This DNA was sheared into fragments averaging approximately 250 bp and then processed for sequencing on an Illumina GAIIx instrument in paired-end format using 100 cycles. The resulting reads were processed using Illumina’s Cassava v. 1.8 software (http://www.illumina.com), then trimmed to a quality standard approximating PHRED Q20 in a sliding window, then all reads shorter than 30 nts or containing any ambiguous nucleotides (e.g., “N”) were eliminated. This retained 108,620,955 reads with an average length of 91.9 nts. These were assembled using a deBruijn graph method within the CLC Genomics Workbench (http://www.clcbio.com) to produce 607 contigs at least 200 nts in length. These were screened separately with tBLASTx [59] with the entire *Physcomitrella patens* mtDNA and cpDNA to identify 15 and 11 candidate contigs for these organelles, respectively, with at least some BLAST E-values of 0. Careful manual examination of these candidates based on length, consistency of matching throughout, and depth of coverage reduced these to six contigs of mtDNA and five contigs of cpDNA in which we had high confidence and which summed, separately, to a rough expectation of each genome size.

To order and orient these contigs and to close any gaps between them, we then produced 40,925 long-read (up to ~8,000 nts) sequences from these same DNA sources on a PacBio RSII instrument using the nanopore-based technology from Pacific Biosciences (http://www.pacificbiosciences.com). These were filtered and broken into subreads by splitting at adaptor sequences using PacBio’s “SMRT” software. We searched these reads using BLASTn with the terminal 300 nts of each of these organelle scaffolds to identify reads that may span junctions between pairs of contigs, and then manually used these reads to order and orient these scaffolds and, in some cases, to supply short stretches of intervening sequences. In the latter case, we then searched back to Illumina reads to identify those that “walk” through the PacBio-only portions to ensure accuracy.

### Gene identification and annotation

Gene and inferred protein sequences of other plant organelle genomes were searched to the *Tetraphis pellucida* mtDNA and cpDNA to identify homologs, first using MAKER [60], then manually for correction and additions. All open reading frames were collected from unannotated regions and searched against GenBank to check for whether any novel genes are present. Protein sequences were inferred assuming the universal genetic code. Careful manual examination was made specifically for the small number of genes inferred here to be missing, but are otherwise present in other bryophyte organelle genomes.

### Prediction of RNA editing sites

The online tool PREPACT 2.0 [32] was used to search for candidate RNA editing sites (C => U) in both the mtDNA and cpDNA assemblies of *Tetraphis pellucida* as well as the published cpDNA of *Tortula ruralis*. The BLASTx prediction method was used with the “commons” option and the default settings. For the mtDNA search the CDS/protein databases used were those for *Chaetospaeridium globosum, Chara vulgaris, Isoetes engelmannii, Lotus japonicas, Marchantia polymorpha, Physcomitrella patens, Selaginella moellendorfii and Silene latifolia* (the same set used for prediction of sites in the *Anomodon rugelii* mtDNA by Lenz & Knoop [32]). For the cpDNA searches the databases were for *Adiantum capillus-veneris, Anthoceros formosae, Chaetospaeridium globosum, Chara vulgaris, Gossypium hirsutum, Marchantia polymorpha, Oryza sativa, Pellia endiviifolia* and *Physcomitrella patens*.

### Availability of supporting data

The data sets supporting the results of this article are available in the NCBI GenBank repository, accession numbers KJ817845 (cpDNA) and KJ817846 (mtDNA).
